# Going beyond (electronic) patient-reported outcomes: harnessing the benefits of smart technology and ecological momentary assessment in cancer survivorship research

**DOI:** 10.1007/s00520-020-05648-x

**Published:** 2020-08-25

**Authors:** Melissa S. Y. Thong, Raymond J. Chan, Corina van den Hurk, Kristen Fessele, Winston Tan, Dagmara Poprawski, Paz Fernández-Ortega, Catherine Paterson, Margaret I. Fitch, MASCC EMA working group

**Affiliations:** 1 Unit of Cancer Survivorship, Division of Clinical Epidemiology and Aging Research, German Cancer Research Center (DKFZ), P.O. Box 101949, 69009 Heidelberg, Germany; 2 Department of Medical Psychology, Amsterdam Medical Centers, location AMC, Amsterdam, Netherlands; 3 School of Nursing, Queensland University of Technology, Queensland, Australia; 4 Cancer and Palliative Care Outcomes Centre, Queensland University of Technology, Queensland, Australia; 5 Princess Alexandra Hospital, Metro South Hospital and Health Services, Queensland, Australia; 6 Department of Research and Development, Netherlands Comprehensive Cancer Organisation (IKNL), Utrecht, Netherlands; 7 Office of Nursing Research, Memorial Sloan Kettering Cancer Center, New York, NY USA; 8 Department of Medicine, Division of Hematology/Oncology, Mayo Clinic Florida, Jacksonville, FL USA; 9 Cancer Service, Limestone Coast Local Health Network, Adelaide, Australia; 10 Institut Català d’Oncologia – ICO, Nursing Research, University of Barcelona, Barcelona, Spain; 11 School of Nursing, Midwifery and Public Health, University of Canberra, Canberra, ACT Australia; 12 ACT Health and Canberra Health Services, Canberra, ACT Australia; 13 Prehabilitation, Activity, Cancer, Exercise and Survivorship (PACES) Research group, University of Canberra, Canberra, ACT Australia; 14 Bloomberg Faculty of Nursing, University of Toronto, Toronto, Canada

**Keywords:** Ecological momentary assessment, Cancer survivors, Repeated measures, Patient-reported outcomes, Real-time data

## Introduction

Rapid developments in digital mobile and sensor technology have facilitated the active and passive collection of detailed, personalized data in increasingly affordable ways [1]. Researchers may be familiar with the daily diary, portable computers, or the pedometer for the collection of patient-reported outcomes (PRO) [2] in cancer survivorship research [3]. Such methods, termed ecological momentary assessment (EMA), have evolved with technological advances, e.g., collecting data or providing interventions (ecological momentary intervention, EMI) via apps or devices such as smartphones [4]. These smart technology–adapted sEMA/sEMI methods are more widely used in affective disorders or addictive behavior research [5, 6] but are currently still under-utilized in cancer survivorship research. A recent scoping review on the use of *active* EMA among cancer survivors identified twelve articles published between 1993 and 2018 [7]. Most of the included studies in that review used portable computers. This commentary will discuss the utility of sEMA/sEMI in cancer survivorship research and call for action to advance this area of science.

## What is EMA?

EMA refers to an intensive method of collecting reports on respondents’ current state [8]. It has also been termed as “experience sampling” or “diary assessment” [8, 9]. Although these terms stress different aspects of EMA, collectively, they encompass methods that involve the “repeated sampling of people’s current thoughts, emotions, behavior, physiological states, and context, in their natural environment, typically (but not necessarily) via electronic wearable devices” [8].

EMA methods can be categorized as being either active or passive. Active EMA requires conscious input from the participants who are prompted to provide information multiple times a day and, typically, over a period of consecutive days [8]. Passive EMA collects observational data through wearable devices/sensors (e.g., pedometers, fitness trackers) without participants’ active involvement [10]. Technological advances in smart devices have broadened the range of data collected with passive EMA tracking. These “digital phenotypes” can be accessed with in-built sensors (e.g., accelerometer, gyroscope, heart rate measurements) or log files data (location tracking, screen activity tracking) [11].

## Why use EMA?

A key feature of EMA is that data is collected in participants’ natural, lived environments (“ecological validity”), as opposed to data collected within a research or laboratory setting [8]. Health behaviors and experiences can be affected by context, and such information is difficult to capture in traditional retrospective assessments. EMA methods have notably furthered the science of substance use, cessation, and relapse, often with results in contrast to theory-driven studies that are largely derived from global reports collected with retrospective questionnaires [12, 13].

In light of the recent movement to implement the use of PRO in clinical routine care [14], including electronically captured PRO (ePRO) via mobile apps [15], it is important to differentiate the nature of clinical use of PRO versus active EMA. Traditional methods of collecting PRO for clinical use tend to rely on global retrospective self-reports, e.g., using a time frame of past 24 h or week. Such measures are often used on a regular basis or immediately prior to clinic visits for pragmatic reasons [16], due to technical/administrative considerations or to minimize possible disruption to clinical workflow [14]. The main drawback of such assessments is recall bias [8]. Active EMA circumvents this problem by asking participants to rate their current state (“momentary”) rather than reflect on aggregated, past experiences. Furthermore, EMA involves repeated assessments at different times per day for consecutive days. This allows the capture of the dynamic changes in symptomatology and behavior or mental states through the interplay with environment, context, social relationships, and time [8]. As an example, cancer-related fatigue could vary depending on time and context [17]. Also, cancer survivors often experience multiple symptoms which can persist indefinitely [18]. EMA could be a less cumbersome and cost-effective alternative to traditional PRO methods in the longitudinal collection of complex multidimensional constructs [10].

## sEMA applications in cancer survivorship

### In clinical setting

sEMA offers significant potential for diagnostic, monitoring, or intervention purposes in the clinical setting.

Active and passive sEMA data can be incorporated in a study to assess effects of diagnosis [19], in active cancer treatment [20], or in a palliative setting. Accelerometer data collected from smartphones could track recovery trajectories such as physical activity following cancer treatment beyond traditional clinical indicators [21] or to support behavioral change [22]. Semantic location data comprising information from smartphone sensors (sound, light, WiFi signals) and global positioning system tracking have explored associations between physical activity and mental health [23].

The advantages of sEMA can also be leveraged in symptom management where continuous monitoring of side effects is of relevance [24]. A novel pilot study uses a package of wireless sensing technologies to collect passive (physiologic and environmental/home ambient factors) and active (subjective experience of pain episode) data that could influence cancer-related pain [25]. Passive sleep monitoring using smartphone sensors offers a potential simpler and cheaper solution to traditional methods to assess sleep problems [26], which is significant in cancer survivors.

An outgrowth of sEMA, sEMI aims to provide interventions remotely in respondents’ everyday lives (i.e., real time) and natural environments. sEMI can be tailored to provide feedback to enhance treatment personalization and adherence, e.g., providing an sEMI following an sEMA on affect or health behavior [27]. Despite a significant increase of e-health/m-health interventions in cancer care [28], assessment of their usage and adherence is not optimal [29]. Integration of sEMA methods such as Just-In-Time Adaptive Interventions can potentially benefit e-health/m-health interventions, [29] as evidenced in studies with psychiatric populations [30] or in weight management [31].

### In research setting

The smartphone is now almost ubiquitous, reducing the need for additional equipment. sEMA could potentially reach populations previously under-researched using traditional data capture methods, e.g., living in remote/rural areas. EMA can collect large amounts of quantitative ideographic (individual) data [10]. Traditional quantitative methods tend to be nomothetic, identifying patterns of behavior across a population of individuals [10]. However, group-level findings may not generalize to the individual [10]. Quantitative ideographic data is especially clinically relevant in the current strive to provide personalized cancer care.

Cancer survivors often experience co-occurring multiple symptoms, e.g., the cluster of sleep problem, fatigue, and depression, of which a challenge exists to identify the “driver” or trigger symptom of these clusters [32]. According to the Network Theory of Psychopathology, recurrent causal loops maintain the “disordered” state (e.g. sleep problem → fatigue → rumination → sleep problem) [10]. These self-sustaining loops could be broken by targeting personalized interventions to the identified patient-specific symptom networks [33]. Identification of such networks require ecologically valid repeated assessments of symptoms, for which EMA is well-suited [10].

## Conclusion

Rapid advances in technology and a marked interest in personalized health care could increase the feasibility and attractiveness of sEMA in cancer survivorship research. Nevertheless, challenges of EMA studies such as reproducibility, comparability of assessment items, and interpretation of results exist [7]. Other concerns such as data provenance and regulatory issues also need to be addressed in light of the growing interest of sEMA in cancer survivorship research [34]. To further advance this field of science and practice, some recommendations include (1) the use of a checklist (e.g., an adapted STROBE checklist) [35] to report ongoing sEMA studies, (2) initiate a Delphi survey to identify areas of focus for the development of best practice guidelines, and (3) the clinical and research community could establish an international interdisciplinary working group similar to the working groups that sought consensus for use of patient-reported outcomes in research and clinical practice [36]. A working group for sEMA could involve expertise representing oncology in clinical practice, research, m-health, implementation science, and consumers to (1) inventory the use of sEMA in cancer survivorship research and to systematically identify potential barriers and solutions of its usage; (2) identify quality hallmarks of apps and platform providers of sEMA in view of the current proliferation; (3) develop guidelines on the design, methodology, and statistical analyses of sEMA studies; and (4) provide guidance on addressing relevant ethical concerns associated with the use of sEMA. Such guidance should include, but not limited to, ethical considerations of using smart sensors/devices; the collection, storage, and sharing of sEMA data [10]; and the benefits versus patient burden in the use of sEMA [37].
